# A Review of Quantitative Evaluation of Electromagnetic Environmental Effects: Research Progress and Trend Analysis

**DOI:** 10.3390/s23094257

**Published:** 2023-04-25

**Authors:** Mei Li, Guanghui Wei

**Affiliations:** 1 National Key Laboratory on Electromagnetic Environment Effects, Shijiazhuang Campus of Army Engineering University of PLA, Shijiazhuang 050003, China; wei-guanghui@sohu.com; 2 School of Information Science and Engineering, Hebei University of Science and Technology, Shijiazhuang 050018, China

**Keywords:** electromagnetic environmental effects, quantitative assessment, objective electromagnetic environmental effects assessment, subjective equipment electromagnetic environmental effects assessment, assessment models, electromagnetic environmental effects assessment system

## Abstract

With the increasing complexity of the electromagnetic environment, existing electronic systems and various equipment are greatly threatened. The study of the assessment of the effects of the electromagnetic environment has attracted more and more attention in the academic field and industry. In this review, the definition of the electromagnetic environmental effects assessment could be classified into two categories: objective and subjective equipment electromagnetic environmental effect assessments. Studies on both are comprehensively reviewed. The electromagnetic environmental effect assessment models are described and analyzed. The effects evaluation models mainly calculate the electromagnetic environmental effect index by various weighting, probability, and normalized interference level methods. However, each proposed model has certain limitations, so it cannot deal with all the electromagnetic interference effects. Therefore, a comprehensive evaluation model for complex electromagnetic environmental effects has not yet been established. Recent studies have shown that neural networks can be used to improve the adaptability of evaluation models in electromagnetic environments. The evaluation result of the normalized interference level method is more consistent with the actual work efficiency. The combination of the two can unify the objective and subjective assessments, which is of great significance to the improvement of the consistency and accuracy of the results of the effects evaluation of the electromagnetic environment. Accordingly, challenges for future research are revealed, which plays a vital role in the development of electromagnetic environmental effects research and provides a reference for researchers.

## 1. Introduction

Studies on electromagnetic environmental effects (E3) can be traced back to the 1930s in the United States. Moreover, a milestone definition of electromagnetic environmental effects was given in the MIL-STD-464 [1] in March 1997. During and after this period, the concept evolved continuously until a nearly consistent definition was given in the JP1-02 [2] in 2008. In China, the concept of electromagnetic environmental effects has been defined in the GJB72A-2002 [3] and GJB1389A-2005 [4] as follows: the influence of the electromagnetic environment on the operation ability of electrical and electronic systems, equipment, and devices. Other countries largely follow the definition of the MIL-STD-464. From that definition, we can see that the study of electromagnetic environmental effects mainly focuses on various objects, which play a crucial role in certain fields [5].

Generally, electromagnetic interference comes from two aspects. On the one hand, static electricity, lightning, and electromagnetic pulse in natural environments [6] will produce electromagnetic radiation. On the other hand, high-power frequency equipment will also generate harmonic interference signals during operation, making the surrounding electromagnetic environment extremely complex [7]. The equipment operating in the electromagnetic environment may have a blocking effect [8], a strong field effect [9], and other complex electromagnetic environmental effects that contribute to the interference. Eventually, this will lead to a loss of work efficiency. In agriculture, the growth of crops in an electromagnetic environment can be hindered [10,11,12]. The human body, when exposed to the electromagnetic environment, will also suffer from radiation [13,14]. Therefore, more and more attention has been paid to the study of electromagnetic environmental effects. This study area has experienced a transition from effect modeling [15,16] to effect evaluation [17] and prediction [18] and from qualitative to quantitative [19,20] study. We believe that the study of the evaluation/prediction of electromagnetic environmental effects should not be limited to effects on a certain object. Even if the object does not exist, the electromagnetic environment may still exist. Hence, we can assess (or evaluate) the electromagnetic environment’s complexity to predict its potential threat to equipment.

For the evaluation, it is crucial to construct a reliable evaluation model to unify the objective and subjective effects and improve the adaptability and accuracy of the evaluation model in various complex electromagnetic environments. This review aims to mainly investigate the evaluation of the effects of electromagnetic environments from two perspectives: the electromagnetic environment and its interference effects on equipment. The modeling methods, input and output evaluation indexes, model performance, applicable conditions, and others related to the evaluation models are comprehensively reviewed. Additionally, future studies are also discovered according to current issues in electromagnetic environmental effects evaluation.

In Section 2, there is an overview of the electromagnetic environmental effects assessment with an illustrated research architecture. In Section 3, we investigate the effects evaluation methods and models of the objective electromagnetic environment to understand the complexity of this environment and the relationship between the electromagnetic environment and the equipment in it. In Section 4, we survey research on the subjective electromagnetic environment. We show the evaluation methods and various evaluation models. In Section 5, we review the development of the effects evaluation systems. In Section 6, we discuss current research issues and future challenges to offer further research references to researchers. In the last section, we conclude this review.

## 2. Overview of Complex Electromagnetic Environmental Effects Assessment

**Figure 1:**
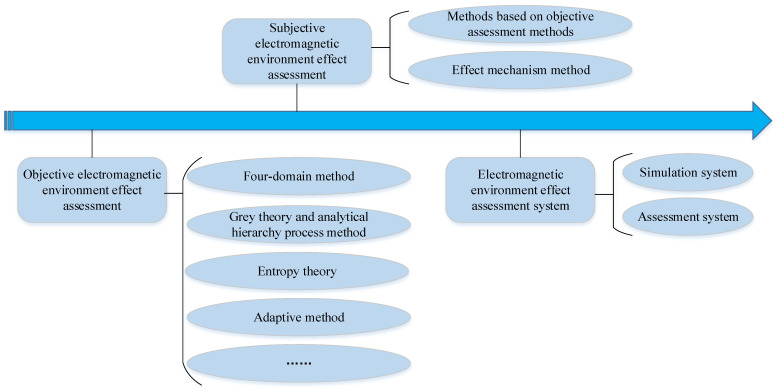
The research architecture of the electromagnetic environmental effects assessment.

In a broad sense, the study of electromagnetic environmental effects refers to research on the complex electromagnetic environment. Not only does it study a specific test object but also the whole electromagnetic environment. That is to say, various definitions of their effects belong to a branch of complex electromagnetic environmental research. The current research is mainly focused on quantitative assessment. According to our extensive literature review, we divide the study of electromagnetic environmental effects into two categories: the assessment of the complex objective electromagnetic environment for a specific environment and the assessment of the effects of the subjective electromagnetic environment for a certain type of equipment (object) [21]. The research architecture of electromagnetic environmental effects assessment is shown in Figure 1.

The objective effects of the electromagnetic environment through an assessment keep a watchful eye on the quantitative evaluation of that environment’s complexity in a specific space, time, and frequency band instead of the equipment’s. However, the subjective assessment effects of the electromagnetic environment are closely related to a piece of equipment. The two types of assessments complement each other. The former usually grades the complexity of the electromagnetic environment according to the equipment’s decreasing work efficiency after interference [22]. Simultaneously, the latter always adopts the methods of an objective effects assessment and then carries out deep research, innovation research, and integration research based on these methods. Although most current research focuses on the electromagnetic environmental effect assessment of subjective equipment, the objective effects assessment still plays a vital role in the development of electromagnetic-related research. To make it easier to evaluate, the electromagnetic environmental effects assessment systems involving previous research findings are developed throughout this review.

## 3. The Objective Electromagnetic Environmental Effects Assessment

### 3.1. Basic Four-Domain Method

**Figure 2:**
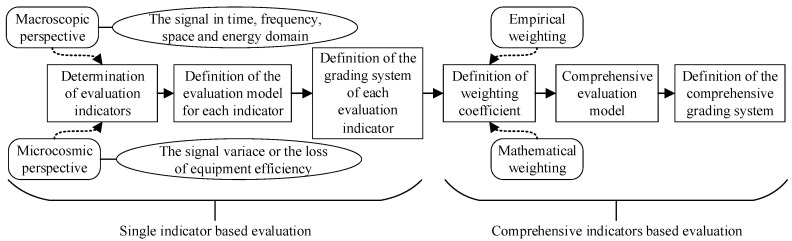
The research design of the four-domain method’s electromagnetic environmental effect evaluation.

The four-domain method refers to time, frequency, space, and energy domains. In the objective quantitative assessment of simple electromagnetic environmental effects, it is necessary to first choose evaluation indexes with macro and micro aspects [23] according to the electromagnetic signal feature in the four domains. In the macro view, more than ten indicators are used to evaluate the complexity of the electromagnetic environment. Generally, researchers adopt signal intensity, background noise intensity, frequency threat degree, frequency occupancy degree, frequency coincidence degree, electromagnetic spectrum control degree, and so on. In the micro view, the work efficiency (WE) of equipment or the changing rate of the relevant macro indexes are used to reflect the complexity of the electromagnetic environment. The comprehensive electromagnetic environmental effects assessment model is established based on the proportion of the resources occupied by the related index exceeding the threshold level of the electronic system. Figure 2 illustrates the research design of the four-domain method for electromagnetic environmental effects assessment.

Dai et al. [24] adopted spatial occupancy (SO), time occupancy (TO), spectrum occupancy (FO), and the average power spectral density (AP) to build the evaluation model. Meanwhile, they used the general evaluation standard [25] to evaluate the electromagnetic environmental complexity from the macroscopic view, and the performance loss of specific equipment was used to define the complexity of the same environment from the microscopic view. The D–S evidence theory and fuzzy mathematics were used to quantify the qualitative result of electromagnetic environmental complexity. It effectively reduces the error of the evaluation.

Based on the traditional TO, FO, and SO, Li et al. [26] combined macro- and micro-assessment factors to quantify the complexity of the electromagnetic environment using the weighting method. For the macroscopic aspect, the statistical law of the electromagnetic environment in sensing data is quantified based on the average energy value (AE), standard deviation (SD), and skewness (SK) of the power spectral density. For the micro aspect, the relative variance ratio (RV), the average variance ratio (AV), and the maximum variance ratio (MV) of the power spectral densities are used to quantify the microscopic changes of the electromagnetic environment. The quantitative evaluation of the electromagnetic environment’s complexity is more refined and accurate. However, the weighting coefficient of each evaluation index is obtained empirically, which will lead to overly subjective models.

Except for the characteristics of the four-domain occupancy degree, this domain’s characteristic correlation degree [27] is also used to evaluate the electromagnetic environmental complexity and its threat [28]. It seems that the four-domain method can comprehensively evaluate the complexity of the electromagnetic environment. However, it has two shortcomings. First, it needs to define the threshold value of the electromagnetic environment. However, there is no unified definition of the threshold; therefore, it is not easy to ensure the reliability of the evaluation results. Second, the weightings in the assessment model are more subjective. If a mathematical weighting is adopted, the iterative calculation process of the weighting is complicated. Therefore, the traditional four-domain models are not optimistic enough in electromagnetic environment evaluations, especially for complex electromagnetic environments. Table 1 shows the commonly used classifications and evaluation criteria for electromagnetic environmental complexity using the basic four-domain method. Where a_1_ to a_3_ and b_1_ to b_3_ are the comprehensive thresholds of the electromagnetic environment in the time, frequency, and airspace domains; AP is the average power spectral density; and S_0_ is the threshold of the electronic system in the energy domain.

### 3.2. Grey Theory and the Analytical Hierarchy Process

**Figure 3:**
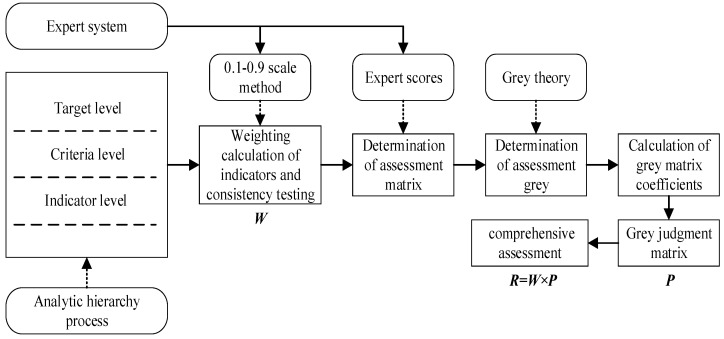
The research design for the electromagnetic environmental effect evaluation is based on grey theory and AHP.

Besides the basic indicators, the analytical hierarchy process (AHP) and grey theory [29] are also used. Although the electromagnetic environment is complex and uncertain, we can still discover some rules in the messy electromagnetic environmental data. Therefore, most researchers employ the grey theory in electromagnetic environmental complexity assessments. Additionally, the AHP is also adopted. The research design for the general electromagnetic environmental complexity based on this method is depicted in Figure 3. AHP is used to model the evaluation system, the weightings are determined based on the expert system and scale method, and the comprehensive evaluation result is obtained using grey theory.

Cui [30] adopted electromagnetic signal density, frequency coincidence, electromagnetic signal types, direction coincidence, and background signal intensity as basic evaluation indicators. They used the fuzzy AHP to build the fuzzy complementary judgment matrix and obtain the corresponding evaluation index weighting. Subsequently, a comprehensive quantitative evaluation model of electromagnetic environmental complexity is constructed.

Gu et al. [31] selected four basic evaluation indexes of the frequency coincidence and frequency occupancy degrees and background and electromagnetic signal densities. Except for the basic signal indexes of the four domains, they also involved the electromagnetic adaptability of a certain type of equipment to reflect the electromagnetic environmental complexity. As a result, they built a comprehensive evaluation model through the weighting method in the same manner as [30]. Additionally, more and more attention is paid to the fusion research [32] of the interference signal characteristics and work efficiency of the equipment in an electromagnetic environment.

Sun et al. [33] evaluated the comprehensive electromagnetic environment’s complexity from the perspective of different objects in that environment. Then, the basic evaluation indexes are determined according to each object. They also used the grey and AHP methods to assess the complexity of the electromagnetic environment, which actualized or realized the unification of qualitative and quantitative assessments.

Dong et al. [34] employed time occupancy, space coverage, spectrum occupancy, average power spectral density, a large signal ratio, a dynamic signal variance ratio, and background signal intensity as the set of basic evaluation indexes of the electromagnetic environment’s complexity. Grey information fusion technology and D–S evidence theory are used in their research. For the improvement of the evaluation resolution and accuracy, a new method for intelligent classification of the electromagnetic environmental complexity is presented.

In the literature review, we can see that grey theory and the AHP can effectively solve the uncertainty problem of the electromagnetic environment. The evaluation system is always modeled at two or three levels, where target and indicator levels are necessary. As shown in Table 2, we can choose the indicators according to the signal, the interference source, the equipment efficiency, the object in the electromagnetic environment, and so on, which suggests that, except for the four domain characteristics (time, frequency, airspace, and energy domains), the electromagnetic behavior characteristics (electromagnetic behavior subject, object, and pattern) are also involved in their environmental complexity evaluation. The model’s performance is always demonstrated by a consistency check (CC) of the index weightings, which should be less than 0.1. The lower the CC, the higher the model’s reliability. A three-tiered evaluation system is better than a two-tiered one due to the combination of objective and subjective evaluations. Additionally, the complexity classification is the same. However, the quantitative threshold value (or range) is expressed differently, which will lead to an inconsistent description of the electromagnetic environment’s complexity. Yet, it still needs to rely on the definition of the electromagnetic environment threshold. Furthermore, the target hierarchy structure, selection of the evaluation matrix, and gray level often follow expert opinion, which is more subjective. Hence, the assessment results are not convincing enough, and the objectivity and accuracy of the assessment model need to be improved.

### 3.3. Entropy Theory

**Figure 4:**
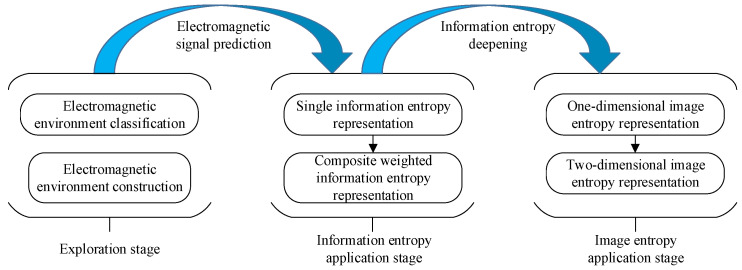
The application of entropy theory in an electromagnetic environmental effects study.

Entropy is a measurement of how chaotic a system is. The messier a system, the greater its entropy. Conversely, the cleaner a system, the lower the entropy. Hence, we can use entropy theory in the electromagnetic environment’s complexity evaluation. The greater the entropy, the more complex the electromagnetic environment, which means that the target object suffers from serious interference from the electromagnetic environment. This method does not need the electromagnetic environment threshold definition in the four-domain models. Some studies [35] show that the entropy theory can also be applied to the assessment-level divisions of the electromagnetic environment. Therefore, it can solve the subjectivity problem in the assessment. Figure 4 illustrates the application of electromagnetic environmental effect research based on entropy theory.

In the exploration research of entropy theory, Zhang et al. [36] proposed an analysis method for building a complex space for the electromagnetic environment through Shannon’s entropy (probability) due to the uncertain characteristics of electromagnetic behavior in that complex environment. It suggests using the entropy method to analyze the complexity of the electromagnetic environment. The entropy theory is also used in the radiation source signal identification [37,38]. These entropy-based methods are preliminary attempts at an objective quantitative analysis of the complexity of the electromagnetic environment.

In their application, Chen et al. [39] proposed a method using compound information entropy to evaluate the electromagnetic environment’s complexity. The probability models of type entropy, density entropy, and distribution entropy are established, respectively. The evaluation indexes in the four domains are involved. Then, the composite information for the entropy model is constructed through the weighting method, which can reasonably illustrate the complexity of the electromagnetic environment. However, each weighting is defined customarily, which leads to subjective models. Shi et al. [40] first calculated the number of quantified gray pixel values at a certain range based on gray image data. Then, the probability of gray pixel values could be obtained. Subsequently, they obtained the image gray distribution entropy based on Shannon’s entropy. The qualitative and quantitative research on both of the two kinds of suppressing interferences, radio and modulated frequency noises, was carried out on ISAR (Inverse Synthesis Aperture Radar) to evaluate the electromagnetic interference. In the initial attempt, the image entropy in the electromagnetic environmental effects evaluation is not refined enough to measure the complexity of the electromagnetic environment. Liu et al. [41] constructed the grayscale image of the electromagnetic environment based on the data in the four domains. They characterized the electromagnetic environment’s complexity through the normalized two-dimensional entropy of the image and expressed this complexity through a hierarchical quantification.

The innovative research of the entropy method avoids the problem of inaccurate evaluation results caused by the definition of the electromagnetic environment threshold value. As shown in Table 3, the utilization of information entropy and image entropy realizes the application of the information method in the electromagnetic environmental effects assessment study. The information entropy method directly uses the data characteristics of electromagnetic signals. Even if the composite information entropy method is adopted to evaluate the electromagnetic environment’s complexity, it remains assessed from different individual information entropy perspectives, and the evaluation results are not compounded. While the image entropy method needs to convert the electromagnetic signal into a gray image, the one-dimensional image entropy method is relatively simple, but the quantitative evaluation results are unclear. The two-dimensional image entropy method seems more accepted, but the evaluation research in a complex electromagnetic environment is worthy of further exploration. Although the entropy method has many drawbacks, it guides the new research field of intelligent electromagnetic environmental effects assessment methods.

### 3.4. Adaptive Evaluation Method

**Figure 5:**
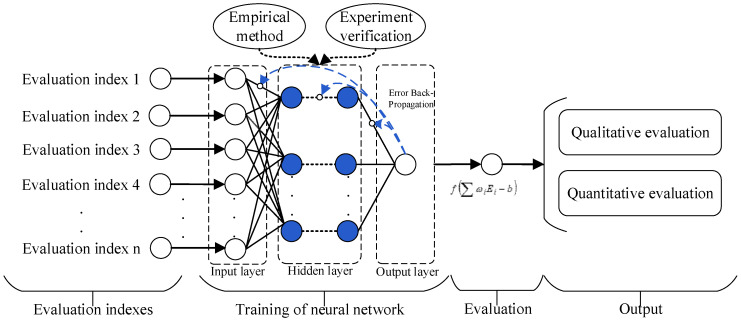
Electromagnetic environmental effect evaluation process based on a neural network.

With the increasing complexity of the electromagnetic environment, more and more evaluation indexes are needed to improve the accuracy of evaluation. Accordingly, the calculation and update of each index weighting will become a crucial issue for future research. Not only will the subjective outcome be obtained by the traditional empirical weighting method, but also complicated calculations will be obtained by the mathematical method. However, adaptive evaluation models based on neural networks can effectively solve the above problems. By constructing neural network models, weightings can be self-trained based on existing data features. Subsequently, an objective evaluation result of electromagnetic environmental complexity can be obtained. Figure 5 illustrates the general evaluation process of electromagnetic environmental effects based on neural networks.

Peng et al. [42] put forward a kind of electromagnetic environmental complexity evaluation method based on feed-forward back-propagation (BP) neural network algorithms adopting the spectrum occupancy (SO) rate, time occupancy (TO) rate, airspace coverage (AC), and background noise intensity (BN). To realize the learning and training of weightings, it also solves the subjectivity and complicated calculation problems of the traditional methods.

Wang et al. [43] believed that various signals show different impacts on the complexity of the electromagnetic environment. Hence, the conventional statistical characteristics of the time, space, frequency, and energy domain indexes may not be able to represent the complexity of the electromagnetic environment. However, a few abnormal activities in significant areas may account for a high proportion of the calculation of the electromagnetic environmental complexity. Based on this, the adaptive fuzzy neural network model is constructed to evaluate the complexity of the electromagnetic environment by using seven indexes: abnormal signal rate (AS), large signal rate (LS), time occupancy (TO), space coverage (SC), channel occupancy (CO), average power spectral density (AP), and background noise intensity (BN).

Dai et al. [44] feed the evaluation indexes of time domain occupancy (TO), frequency domain occupancy (FO), energy occupancy (EO), frequency overlap (FP), signal modulation format (SM), channel occupancy (CO), and background noise intensity (BN) of electromagnetic signals to the extreme learning machine integration model to evaluate the electromagnetic environment’s complexity. This method also belongs to the category of BP neural networks.

Typical research with adaptive methods is shown in Table 4. The adaptive intelligent method uses the testing data of the electromagnetic environment in four domains, extracts the characteristics of the data as network input, and trains the evaluation model parameters based on a neural network. The key is to determine the number of hidden layers and the number of hidden layer neurons. Overcomplicated hidden layer structures may lead to overfitting of the model, while oversimplified hidden layers may lead to underfitting. Therefore, we should select the appropriate network structure. Single hidden layer networks can use the cross-verification method [42], experimental methods (incremental and pruning methods) [44], and others. The fuzzy interference method [43] can be adopted for complex network structures. Although the neural network method can adaptively adjust the model weightings to obtain relatively objective results, there are still some problems in the current research. First, the limited amount of electromagnetic environment data may weaken the model’s performance. Second, the training and verification are carried out in the same electromagnetic environment, so the application ability of the model in other electromagnetic environments is worthy of further discussion. Third, the proposed models are based on basic neural network methods, which makes them difficult to apply well to other strong nonlinear evaluation methods.

In addition to the above assessment methods, there are also system dynamic methods [45], exponential methods [46], game theory methods [47], and others in the quantitative assessment of objective electromagnetic environmental effects. Generally, regardless of the methods, the above all need to classify the electromagnetic environment into four levels: slightly, mildly, moderately, and severely. Besides this, it also comprehensively determines the complexity of the electromagnetic environment in four domains. In practice, more evaluation indicators are involved, and each indicator needs to be weighted to evaluate the comprehensive electromagnetic environmental effects. In these studies, it is crucial to obtain more accurate weighting coefficients. In some studies, each level can be refined and divided into several internal levels to make the evaluation more accurate. Although the evaluation criteria at each level are similar, there is no uniform and strict definition of this value. Moreover, these studies mainly quantify the evaluation effects of an electromagnetic environment and pay less attention to the adaptability of the research of the equipment in the electromagnetic environment. However, the electromagnetic environmental effects assessment for the specific equipment is more meaningful in practice [32].

## 4. Effect Evaluation of Electromagnetic Environment on Equipment

According to the research connotation of electromagnetic environmental effects [48], the assessment of these effects on subjective equipment is to evaluate the subjects’ tolerance to electromagnetic interference. When we place different test equipment in the same objective electromagnetic environment, their anti-interference abilities may be distinct. Therefore, the research on the electromagnetic environmental effects assessment of equipment has more practical value. We can divide the subjective electromagnetic environmental effects assessment methods into two categories: commonly used methods and effect mechanism methods.

### 4.1. Commonly Used Methods

In the subjective equipment electromagnetic environmental effects assessment, the general objective assessment methods are adopted too. However, the survival probability [49] of the test equipment in the electromagnetic environment should also be considered.

Wei et al. [50] proposed a quantitative assessment method for the electromagnetic environmental effects of airborne avionics systems. The electromagnetic sensitivity test data of the equipment is taken as the evaluation reference. The multi-layer fuzzy comprehensive evaluation theory is used as the evaluation method, and the D–S (Dempster–Shafer) evidence theory is employed to process the interference value between the safety margin curve and the sensitivity threshold in the electromagnetic sensitivity test. The critical categories of subsystems and equipment in the national military standard GJB72A-2002 are used to determine the weights. It provides a new way to solve the problem of the electromagnetic environmental complexity evaluation of avionics systems.

Shu [51] used the index calculation method based on a fuzzy set and the AHP to study the electromagnetic radiation effects on a vehicle’s electronic control system in an electromagnetic pulse environment. Jiang et al. [52] believed that useful signals and critical sensitivity played a vital role in the electromagnetic environmental effects evaluation of the equipment. They built an electromagnetic damage effect evaluation model for a ship’s electronic equipment by combining the AHP with the comprehensive fuzzy evaluation method. The system’s electromagnetic damage degree was divided into five levels to show the equipment performance of the ship’s electrical system. In theory, the comprehensive evaluation performance of this model is better. Although it lacks experimental verification and cannot obtain the actual evaluation effect of the model, the application of useful signal and critical sensitivity in this model has great significance for the evaluation of electromagnetic environmental effects. Han [53] proposed an algorithm combining fuzzy clusters to find the optimal interference waveform that could cause damage to radio fuses. In the mentioned models, multiple indexes [54,55] are closely related to the test equipment involved. The accurate weighting coefficients of these indexes are always difficult to calculate due to subjective judgment [56], and more performance indicators of the test equipment usually need to be obtained. Not only is the calculation complicated, but also their application is limited.

When referring to the application of artificial neural networks in electromagnetic radiation effect predictions, Li et al. [57] took the electromagnetic interference parameters as the input of the neural network. They established the prediction model with the error backpropagation neural network, which effectively solves the problem of the crosstalk effect between the wires. Ellithy et al. [58] proposed a method based on artificial neural networks to predict the electromagnetic interference effect of high-voltage transmission lines and public gas transmission pipelines. Subsequently, they took the natural gas pipeline in Oman and Sudan as the test object to verify the effectiveness of this method. Based on the above methods, the researchers explored fusion evaluation methods such as the grey analytic hierarchy process combined with BP neural networks [59], fuzzy theory combined with neural networks [60], and others.

The commonly used evaluation methods for equipment are shown in Table 5. According to the literature review, we can divide them into three categories: AHP and fuzzy theory-based methods, neural network-based methods, and fusion methods. The AHP- and fuzzy theory-based method is adopted for effect evaluation on equipment. When a neural network is involved, the model can be used for prediction. In the fusion method, while the AHP and fuzzy theory are employed to generate the input data of the neural network, this network is used to enhance the model’s nonlinear capability. They have two shortcomings: First, multiple indexes related to the specific equipment in the above models will result in a poor generality of each model. Second, limited test data for an electromagnetic environment will inhibit the application of neural network-based models. In addition, the sensitivity threshold of the test equipment is also involved in the evaluation and prediction, which demonstrates that it is more crucial for evaluation or prediction. We can obtain two points from these studies. First, the sensitivity threshold is more crucial to the evaluation of the effects. Second, the fusion methods may be a reference for improving the model’s accuracy.

### 4.2. Effects Mechanism Methods

**Figure 6:**
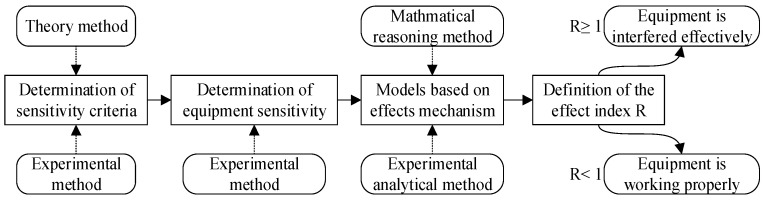
The research design of effect mechanism methods.

The effect mechanism method directly analyzes the interference on the equipment physically. Electromagnetic interference can not only enter the system by way of “front door coupling” [61] through the antenna but also enter the system by way of “back door coupling” [62] through the shell, cables, and holes in the chassis to damage its internal components. Both interference mechanisms are distinct. Therefore, it is more consistent with the characteristics of equipment subjected to electromagnetic interference to analyze the electromagnetic environmental effects of various equipment [63,64] based on the effects mechanism. This method will construct the effect evaluation model through mathematical reasoning and experimental analysis. However, the sensitivity threshold of the equipment is usually necessary to define the critical interference of the model. Hence, the definition of sensitivity criteria is an essential precondition for effect evaluation models. The research design of the effect mechanism methods is shown in Figure 6. In these methods, the interference index of R will be defined. When R ≥ 1, the equipment has interfered; when R < 1, the equipment is working properly.

#### 4.2.1. Sensitivity Criteria

The effect mechanism method establishes the effect’s evaluation model by analyzing the interference weighting of different interference components on the equipment. Hence, it first determines the sensitivity threshold of the equipment, both in-band and out-of-band. Subsequently, the reliability of the model is verified by the effect evaluation results of the critical interference. It means that the sensitivity criterion is of great significance to the electromagnetic environmental effects evaluation using this mechanism’s method. It is also a primary problem to be solved in future research. Generally, the interference margin [65] can be employed as the sensitivity criterion for most electronic equipment. Digital communication systems always use the interference level [66] or bit error rate [67] as the sensitivity criterion. UAV (Unmanned Aerial Vehicle) information link [68] adopts AGC voltage and bit error rate as its sensitivity criteria to analyze the electromagnetic interference effect of a data link. In the sensitivity criterion of communication radios [69], the work damage level can be used as the sensitivity criterion for the voice communication system, and 10% of the fixed bit error rate can be adopted for the digital communication system. However, in the following research on digital communication radio [70], it was found that a fixed bit error rate can no longer be taken as the sensitivity criterion of communication radio under electromagnetic pulse-string interference. Their findings show that the proportional relationship between the sensitive bit error rate and the pulse repetition rate is more accurately taken as the sensitivity criterion. Therefore, it is necessary to take the type of interference signal into account to obtain a more precise sensitivity criterion. The radar uses the peak echo level compression of the measured target [71] as the sensitivity criterion. The compression amount varies with the interference threshold. To solve the universality problem of the radar sensitivity criterion, the researcher conducted a further experimental study [72] on it. They found that using an effective interference probability of 40–60% as the radar sensitivity criterion could be better. For the research on the sensitivity criterion of the navigation receiver, Zhang [73] took the satellite’s CNR of 30 dB-Hz as the sensitivity criterion of the navigation receiver. However, we carried out dozens of experiments [74] to prove that it is unreliable to take the CNR as the sensitivity criterion of the navigation receiver. In experiments, the CNR of each satellite without interference and that under critical interference are disparate. It shows that taking 30 dB-Hz as the sensitivity criterion is not the best choice. We verified that taking the continuous loss of positioning for 30 s as the sensitivity criterion is more accurate when the navigation receiver is subjected to critical interference. The sensitivity research is shown in Table 6.

According to the literature analysis and experiments, we can see that the sensitivity criteria used are also diverse for different equipment. Even the same equipment has distinct sensitivity criteria due to the disparate experiment methods. In the current studies, the single-frequency continuous wave is mainly used as the interference signal to build the electromagnetic environmental effect test platform. Injection and radiation methods are always used to generate interference signals. In theory, they could obtain the same testing results. However, the test results of the former are more theoretical due to the pure injection signal. That is to say, the test results of the latter are closer to the actual working of the equipment. In brief, the most precise sensitivity criterion should be determined through the experiment. It is also necessary to have further studies on sensitivity criteria to obtain consistent sensitivity testing results for the same equipment.

#### 4.2.2. Evaluation Models

**Figure 7:**
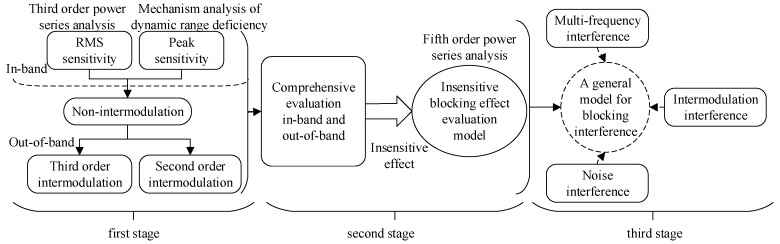
The research process for the evaluation models is based on the effect mechanism method.

Figure 7 demonstrates the research process of the evaluation models based on the effect mechanism method. In the first stage, we only tackled the essential effect evaluation issues, such as the intermodulation and non-intermodulation blocking effects, separately. However, with the deepening of the research process, we are encountering or will encounter new interference effects in the second stage. Therefore, we need to construct models to solve the new problems of effect evaluation. In our current research, we have established more than one model to solve various effect evaluation problems. However, some of the equipment may have multiple effects. In this case, using different effect evaluation models on the same equipment will lead to inconsistency in the evaluation results and a cumbersome evaluation. We should simplify the evaluation models in the following studies. In other words, we expect to obtain a new model involving more interference effects, such as multi-frequency interference, intermodulation interference, and noise interference.

Based on previous studies, a large amount of research has been carried out in recent years. Li et al. [75] found that the blocking interference phenomena of radio stations can be divided into two categories through the electromagnetic environmental effects experiment. Based on the third-order power series expansion analysis, they built two different effects evaluation models: the RMS (Root Mean Square) sensitive model and the peak-level sensitive model. They did so by analyzing the blocking interference mechanisms of equipment radio frequencies. They accurately evaluated the effect of in-band blocking interference of communication stations. According to the literature [75], Wang et al. [76] mainly assessed the effects of out-of-band blocking interference from radio stations. They found that the effect evaluation models of in-band blocking interference are also applicable to out-of-band blocking interference, which indicates that the non-intermodulation interference mechanism of out-of-band is the same as that of in-band. However, a completely different phenomenon was found in the out-of-band interference in the electromagnetic environmental effect test. Researchers confirmed that it belongs to intermodulation interference through experiments and theoretical analysis. Subsequently, they built the second-order [77] and third-order [78] intermodulation effect evaluation models, respectively. The reliability of their models is verified, and their mechanism is also illustrated.

The above effects evaluation models are all based on the experiment on the communication radio station. Is this a unique property of a communication radio station or a property shared by all kinds of equipment? To verify if the above models are also applicable to other equipment, Zhao et al. [79] and Wei et al. [80] employed the third- and second-order intermodulation effects evaluation models on a navigation receiver. The effect evaluation error under critical interference is less than 1 dB, which demonstrates the excellent applicability of the third- and second-order intermodulation models.

In studies, the multi-frequency interference effects model is also used to evaluate the noise interference effect. Based on the non-intermodulation blocking effect evaluation model [75], Li et al. [81] found that the model is unsatisfactory on the navigation receiver and that the existing model based on the third-order power series expansion has shortcomings. It is necessary to make further improvements to current models. In the experiment of multi-frequency non-intermodulation electromagnetic radiation effect on radar, Zhao et al. [71] adopted peak-level compression of the target echo as the blocking sensitivity criterion to evaluate the electromagnetic environmental effect. They found a new phenomenon named DIE (Dual-frequency Insensitive Effect). Theoretically, the fifth-order term of the nonlinear system is the root reason for the new interference effect. To solve the problem of assessing the electromagnetic environmental effects of equipment with insensitive effects, we built the assessment model [82] of the insensitive blocking effect based on the effect mechanism. The model verification was carried out on an insensitive radar and a sensitive communication radio station. On the one hand, the model’s effectiveness is examined by the dual-frequency electromagnetic radiation effect test. On the other hand, the universality evaluation model research of the electromagnetic environmental effect is explored.

The analysis of evaluation models based on effect mechanism analysis is shown in Table 7. Each evaluation model is appropriate for each kind of effect phenomenon. However, all evaluation models are based on the normalized interference level, $E_{i}/E_{i0}$. In different interference effects, additional parameters will be introduced to adjust the models, such as the blocking interference factor, the normalized factor, and others. The ratio of amplitude wave level to sinusoidal continuous wave level, $E_{ame}/E_{\sin e}$, is always adopted to simplify the evaluation model. In brief, the evaluation model based on effect mechanism analysis mainly considers the contribution of each interference component to the total interference with the normalized interference level, which provides evidence for the general model.

## 5. Electromagnetic Environmental Effects Evaluation System

The effects evaluation system of the electromagnetic environment takes significant effort after constructing the evaluation model; it supplements the evaluation study. Besides a significant reduction in the calculation, the accuracy is also improved. With the development of the effects evaluation system of the electromagnetic environment, the effects assessment research is more useful for engineering applications.

In 1968, W.R. Johnson and A.K. Thomas first proposed a computer-aided analysis method to solve electromagnetic compatibility problems. In the 1970s and 1980s, the United States and Russia developed EMI prediction software to meet different engineering demands [83]. IEMCAP (In-system Electromagnetic Compatibility Analysis Program) and SEMCAP (Inter-system Electromagnetic Compatibility Analysis Program) are the earliest prototypes [84,85] of electromagnetic compatibility prediction programs developed in the United States, respectively. After that, the ROM Aeronautical Development Center of the United States developed IPP-1 (Interference Prediction Program One) software for radar systems. Subsequently, the MSC Corporation of the United States developed ARIES software for 3D solid modeling, EMAS software for EMI/EMC analysis, and UWAVELAB software for high-frequency electromagnetic analysis [86,87]. The Boeing Company in the United States also has its own electromagnetic compatibility prediction software but has not launched it due to confidentiality.

In China, the Beijing University of Aeronautics and Astronautics first developed EMC prediction software for aircraft EMC prediction in the 1990s. Chen [88] studied the prediction of electromagnetic compatibility between radio systems. He also developed software for electromagnetic compatibility prediction, analysis, and simulation between systems. Based on the testing data, Chen et al. [89] comprehensively used the fitting model and prediction algorithm to predict the electromagnetic compatibility between the communication systems and implemented the software with .NET. Zhao et al. [90] adopted electromagnetic topology and power balance methods to evaluate the high-frequency response of the cavity, and they developed software for simple simulation. Li et al. [91] built a model of significant electromagnetic parameters with testing data that could predict the variation of the electromagnetic environment in a specific region; a simple electromagnetic environment analysis software was also developed. Zeng et al. [92] developed software to analyze and predict complex electromagnetic environments. Li et al. [93] developed electromagnetic environment adaptability assessment software for frequency equipment according to the theoretical radiation effect model of communication equipment and the effect testing data of communication equipment. Yang et al. [94] analyzed the impact of a spacecraft’s electric propulsion plumes on a satellite’s electromagnetic environment. Besides this analysis, they developed electromagnetic effect simulation software to provide a reference for reducing the effects evaluation cost of space systems.

In summary, there is not much research on the actual electromagnetic environmental effects evaluation system. The current electromagnetic environmental effects evaluation software is mainly for simulation, and the software used for prediction is more limited to a single source and frequency of interference. The related software does not involve the correlation between multiple sources and frequencies of interference. Hence, the application of this kind of software in a complex electromagnetic environment faces a big challenge.

## 6. Discussion and Future Challenges

The electromagnetic environment is becoming increasingly complex, and the vital role of its effect evaluation research has become prominent. The study of objective electromagnetic environmental effects takes the characteristics of the four-domain electromagnetic environmental signals as the research object. There are also a small number of relevant studies that reflect on the interference threat posed by the electromagnetic environment’s complexity on equipment. It inspires us to evaluate the electromagnetic environmental complexity from the perspective of individual equipment, allowing for the unification of objective and subjective assessments.

The evaluation of complex electromagnetic environmental effects needs to be analyzed from the perspective of multi-source interference. Although the complex electromagnetic environment is mentioned in current research, it mainly analyzes it from the perspective of the multiple characteristics of interference signals in four domains. It does not really involve the electromagnetic environmental effects under the action of multi-source signals.

For the effects evaluation of the electromagnetic environment combining the characteristics of interference signals and the work efficiency of the test equipment, the indexes of the environmental effects signal are mainly determined manually; however, the selection of indexes is different and the model adaptability is poor. The subjective evaluation in terms of the effects mechanism analysis is mainly based on interference levels and equipment sensitivity. The evaluation indexes are consistent with each other, but the experimental environment has a significant impact on those results.

Although the BP neural network is introduced to train model weightings and the model’s parameters are processed based on AHP and other methods, the input parameters of the neural network are still determined manually, leading to greater subjectivity of the evaluation indicators. These indicators, which are closely related to the evaluation of the electromagnetic environment and equipment, may be missed.

The tremendous amount of research is replete with the efforts of many researchers for the electromagnetic environmental effects evaluation, indicating that it has a solid theoretical foundation and engineering application. Based on large amounts of analysis of the literature on the effects evaluation of the electromagnetic environment and our contributions to research, some research ideas are worthy of further exploration and inquiry as follows.

### 6.1. The Comprehensive Subjective Equipment Evaluation Model Based on Effects Mechanism Analysis

In current research, several kinds of evaluation models aim to capture the different effects of electromagnetic radiation on equipment. Evidently, a quantitative evaluation method adopting a single evaluation index simplifies this model to a certain extent. In addition, the equipment is an indivisible whole, so it is one-sided to evaluate the electromagnetic environmental effect on the performance of equipment from any independent model, which cannot fully reflect the anti-jamming ability of the equipment. Therefore, it is very urgent to construct a comprehensive evaluation model for equipment that integrates multiple interference effects (multi-frequency interference, noise interference, and third-order intermodulation interference) based on the analysis of the cross-linking relationships of each independent model in the study of effect mechanisms. On the one hand, it will simplify the engineering application of the interference effect evaluation with a few evaluation indexes; on the other hand, it can comprehensively evaluate the electromagnetic environmental effects on equipment. Meanwhile, it is also a crucial research direction to examine the universality of the effects evaluation models.

### 6.2. Adaptive Intelligent Evaluation Model Adopting Deep Learning

According to the above literature analysis, we can see that the application of the neural network to the effects evaluation model of the electromagnetic environment has been reported. However, the performance of the simple neural network-based model is not satisfactory due to the following reasons: a lack of standard electromagnetic environmental data, a small amount of testing data on the electromagnetic environmental effects, limited features of the data for learning, and other reasons. The deep learning method makes the model more adaptive, whereas the effect mechanism method is more in line with the actual interference effect. Subsequently, the effect mechanism and deep learning methods can be effectively combined; thereby, the basic model is built based on the effect mechanism method, and the model parameters are updated by the deep learning method to improve the quality and accuracy of the effect evaluation through the information fusion method. Although we can follow these steps to build the combination model, we still face challenges. The deep learning method requires a great deal of training data to achieve reliable training results through continuous data accumulation. In addition, a standard electromagnetic environmental effect evaluation database is necessary to verify the reliability of the proposed models.

### 6.3. The Complex Electromagnetic Environmental Effects Evaluation System

Generally, the current software for electromagnetic environmental effects research is mainly for simulation modeling. Evaluation and prediction software is more limited to a single source and frequency of interference, as well as a specific space and equipment. The evaluation of the electromagnetic environmental effects of any equipment under the combined action of multiple sources and frequencies is not available. Therefore, it is not suitable for evaluating the interference effects of a complex electromagnetic environment. The evaluation system for the electromagnetic environmental effect is the convergence point of previous studies on the effect evaluation model. This model is the basis of the evaluation system. Therefore, building a comprehensive evaluation model for the complex electromagnetic environmental effects is key to the evaluation system. However, the evaluation model for complex electromagnetic environmental effects is bound to be more complicated than that for a single frequency and source of electromagnetic environmental effects. Hence, it is necessary to simplify the model from an engineering perspective and improve its adaptability [95]. Table 8 shows the possible solutions and key issues for each challenge.

## 7. Conclusions

The accuracy and applicability of the electromagnetic environmental effects evaluation models have always been important parts of this research area. The effects evaluation models mainly calculate the electromagnetic environmental effect index by weighting the contribution of each evaluation index to the effect, including simple weighting, fuzzy hierarchy weighting, BP neural network weighting, statistical, and normalized interference level methods. Simple weighting and fuzzy hierarchical weighting methods are feasible to evaluate the effects in specific electromagnetic environments. Neural networks are the best choice to improve the adaptability of the proposed models in various complex electromagnetic environments. The normalized interference level method applies to the effects evaluation of equipment. However, each proposed model has certain limitations, so it cannot deal with all electromagnetic interference effects.

The combination of objective and subjective evaluation methods is necessary to realize the unification of both evaluations. The combination of the effect mechanism method and deep neural networks can not only meet the requirement of evaluation accuracy but also solve the model’s poor adaptability.

In this paper, the studies and evaluation of the effects of the electromagnetic environment are analyzed systematically, especially for the evaluation models. Both the objective and subjective evaluation models illustrated in this review demonstrate the significant role of electromagnetic environmental effects in certain fields. More research and study efforts are needed to improve the generality, adaptability, and evaluation convenience of the evaluation models.

## Figures and Tables

**Table 1:** Typical comprehensive evaluation based on the basic four-domain method.

Main Method	Evaluation Indexes		Complexity Grade and Evaluation Criteria		Weighting
	Macro Aspect	Micro Aspect	Macro	Micro	
Method 1 [24]	TO, FO, SO, AP	WE	C = TO × FO × SO Slightly: C ≤ 0.1 and AP ≤ 0.5 S_0_ Mildly: 0.1 < C ≤ 0.3 and 0.5 S_0_ < AP ≤ S_0_ Moderately: 0.3 < C ≤ 0.5 and S_0_ < AP ≤ 2 S_0_ Severely: C > 0.5 and AP > 2 S_0_	Slightly: WE ∈ [0, 0.1) Mildly: WE ∈ [0.1, 0.4) Moderately: WE ∈ [0.4, 0.8) Severely: WE ∈ [0.8, 1.0)	×
Method 2 [26]	TO, FO, SO, AE, SD, SK	RV, AV, MV	C = 0.4 (TO × FO × SO) + 0.1 AE + 0.1 SD + 0.1 SK + 0.1 RV + 0.1 AV + 0.1 MV Slightly: 0 ≤ C < 1 Mildly: 1 ≤ C < 4 Moderately: 4 ≤ C < 7 Severely: 7 ≤ C ≤ 10		√
Method 4 [27]	TO, FO, SO, AP	TR, SR, FR, ER	C = ${\left(TO\times FO\times SO\right)}^{\frac{1}{3}}$ Slightly: 0 < C ≤ 0.1 or AP ≤ b_1_S_0_ Mildly: 0.1 < C ≤ 0.4 or b_1_S_0_ < AP ≤ b_2_S_0_ Moderately: 0.4 < C ≤ 0.7 or b_2_S_0_ < AP ≤ b_3_S_0_ Severely: 0.7 < C ≤ 1.0 or AP ≥ b_3_S_0_	T = f(TR•SR•FR•ER) Slightly: T ≤ S_0_ Mildly: S_0_ ≤ T ≤ a_1_S_0_ Moderately: a_1_S_0_ ≤ T ≤ a_2_S_0_ Severely: a_2_S_0_ ≤ T ≤ a_3_S_0_ Work damage: T ≥ S_max_	√

**Table 2:** The typical hierarchy of the evaluation system and quantitative representation in grey theory and the AHP method.

Electromagnetic Environment	The Hierarchy of the Evaluation System	Complexity Classification and the Quantitative Value	Reliability of Index Weighting (CC)
Environment 1 [30]	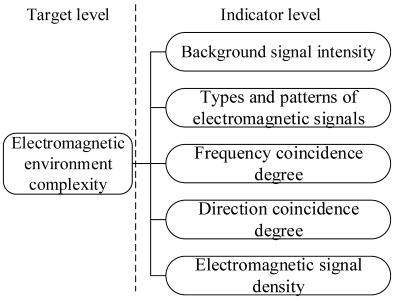	Simply: 0–0.2 Mildly: 0.2–0.4 Moderately: 0.4–0.7 Severely: 0.7–1	CC = 0.098 < 0.1
Environment 2 [31]	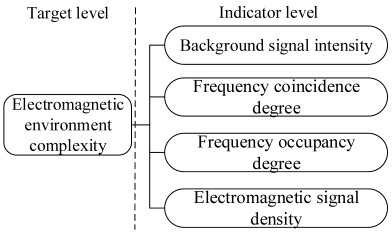	Simply: 0–(10 + 10K)% Mildly: (10 + 10K)%–(30 + 5K)% Moderately: (30 + 5K)%–(60 + 2K)% Severely: >(60 + 2K)% K is the adaptability coefficient of a specific equipment in the electromagnetic environment	-
Environment 3 [33]	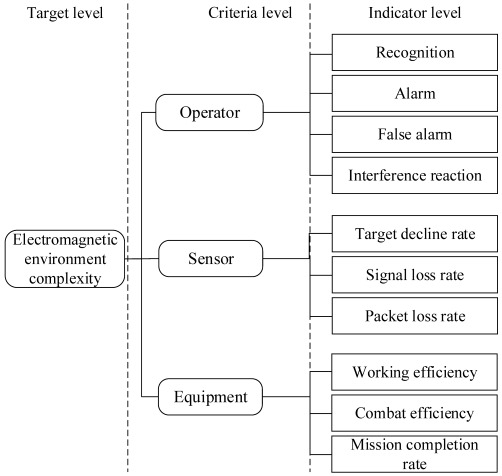	Simply: 3 Mildly: 5 Moderately: 7 Severely: 9	CC = 0.056 < 0.1

**Table 3:** The typical complexity representation of an electromagnetic environment with entropy theory.

Entropy Theory	Qualitative Assessment	Quantitative Standard	Complexity Representation of Electromagnetic Environment	Entropy Value
Composite information entropy [39]	-	-	Type entropy Density entropy Distribution entropy	$H_{1}=-K_{1}\sum_{i=1}^{n1}P(Si)\mathrm{lg}P(Si)$$H_{2}=-K_{2}\sum_{i=1}^{n2}P(Fi)\mathrm{lg}P(Fi)$$H_{3}=-K_{3}/\sum_{i=1}^{ni}\sum_{j=1}^{nj}P(Dij)\mathrm{lg}P(Dij)$ H = H_1_ + H_2_ + H_3_
One-dimensional image entropy [40]	Slightly Moderately Severely	-	Pixel gray value	$H=-\sum_{i=1}^{n}p_{i}\mathrm{lg}p_{i}$
Two-dimensional image entropy [41]	Simply Mildly Moderately Severely	0.0–0.4 0.4–0.6 0.6–0.8 0.8–1.0	Pixel gray value and Image neighborhood gray mean value	$H=\frac{-\sum_{i=0}^{n}\sum_{j=0}^{n}P_{ij}\mathrm{lg}P_{ij}}{H_{\max}}$

**Table 4:** The research on complexity evaluation with an adaptive method.

Method	Evaluation Indexes	Hidden Layers	How to Build the Network Structure?	Training Method
BP neural network [42]	SO, TO, AC, AP	1	Cross-validation	Levenberg–Marquardt (L–M) method
Adaptive neural fuzzy inference [43]	AS, LS, TO, SC, CO, AP, BN	3	Subtractive clustering and experiment	Fuzzy inference method
Machine learning [44]	TO, FO, EO, FP, SM, CO, BN	1	Incremental method, pruning method, and experiment	-

**Table 5:** The commonly used methods on equipment.

Function	Equipment	Methods					Comprehensive Evaluation Index	Performance
		D-S Theory	AHP	Fuzzy Theory	Sensitivity Threshold	Neural Network		
Evaluation	Avionics systems [50]	√	√	√	√	-	Failure probability of equipment	Evaluation probability improved by 15.7%
	Vehicle subsystems [51]	-	√	√	-	-	Shielding efficiency of the shell	CC = 0.017 < 0.1
	Electronic system of ship [52]	-	√	√	√	-	Electromagnetic damage resistance probability	-
Prediction	Crosstalk coupling between two wires [57]	-	-	-	√	√	Peak level of coupling voltage	Evaluation error < 2%
	Gas pipeline [58]	-	-	-	√	√	Induced voltage on the gas pipeline	High accuracy
	Data-link systems [59]	-	√	√	-	√	Efficiency of data link	Precision = 1 × 10^−5^, iteration = 100
	Vehicle cable [60]	-		√	√	√	Peak level	Precision = 4.34 × 10^−5^, iteration = 250

**Table 6:** The sensitivity criterion research on equipment.

Test Equipment	Sensitivity Criteria	Interference Signal		Test Method		Error
		Single-Frequency Continuous Wave	Others	Injection Method	Radiation Method	
Voice communication radio [69]	Work-break level	√	-	-	√	<1 dB
Digital communication radio [69]	Bit Error Rate of 10%	√	-	√	-	<1 dB
Digital communication radio [70]	The relationship between the BER and the pulse repetition	-	Electromagnetic pulse	√	-	-
Radar [71]	Peak echo level compression	√	-	-	√	-
Radar [72]	Effective jamming probability of 40–60%	√	-	-	√	<1 dB
Navigation receiver [73]	CNR of 30 dB-Hz	√	-	√	-	-
Navigation receiver [74]	Loss of positioning within 4 s and lasts 30 s	√	-	-	√	-

**Table 7:** The evaluation models are based on effect mechanism analysis.

Evaluation Models	Methods	Parameters		Definition of Sensitive Types	Application	Evaluation Error (dB)
		Basic Parameters (Normalized Level)	Others			
RMS sensitive model [75]	Third-order power series analysis	$\frac{E_{i}^{2}}{E_{i0}^{2}}$	-	$\frac{E_{ame}}{E_{\sin e}}=1$	Weak nonlinear systems	<1 dB
Peak-level sensitive model [75]	Effect mechanism analysis of insufficient dynamic range	$\frac{E_{i}^{}}{E_{i0}^{}}$	-	$\frac{E_{ame}}{E_{\sin e}}=0.7$	Infinitely strong nonlinear systems	<1 dB
Second-order intermodulation model [77]	Field path coupling principle, third-order power series analysis	$\frac{E_{1}}{E_{10}}$ $,\;\frac{E_{2}}{E_{20}}$	Second-order intermodulation blocking interference factors: $\beta_{1}$ and $\beta_{2}$; low frequency interference relative level: $L_{r}$	The generated interference signal $f=f_{1}-f_{2}$ is in working frequency bandwidth of equipment	Intermodulation interference out-of-band	<10 dB
Third-order intermodulation model [78]	Field path coupling principle, third-order power series analysis	$\frac{E_{00}}{E_{f0}}$ $,\;\frac{E_{1}^{2}}{E_{10}^{2}}$ $,\;\frac{E_{2}}{E_{20}}$	Third-order intermodulation blocking interference factors: $\alpha_{1}$ and $\alpha_{2}$	The generated interference signal $f=\left(2f_{1}-f_{2}\right)$ is in working frequency bandwidth of equipment	Intermodulation interference out-of-band	<3 dB
Insensitive model [82]	Fifth-order power series analysis	$\frac{E_{i}^{2}}{E_{i0}^{2}}$	Correction factor: Δ; normalized coefficient: η	Disappearance of interference on equipment with the increasing interference signal	Strong nonlinear systems	<1 dB

**Table 8:** The future challenges and possible solutions.

Future Challenges	Basic Methods	Possible Solutions	Key Issues
Comprehensive evaluation model	Independent effect mechanism models	Analysis of the cross-linking relationships among independent evaluation models → parameters definition of the general evaluation model → construction of a comprehensive model involving multi-effects → definition of the effect index	Analysis of the cross-linking relationships among independent evaluation models
Adaptive intelligent evaluation model	Effect mechanism models and deep learning	Construction of effect evaluation models based on the effect mechanism → model parameters training based on the deep neural network → nonlinear transformation with activation functions → model update	A great deal of training data is necessary
Complex electromagnetic environment evaluation system	Effect evaluation model under a complex electromagnetic environment	Construction of an effect evaluation model under a complex electromagnetic environment → engineering simplification of the model → implementation	Construction of an effect evaluation model under a complex electromagnetic environment

## Data Availability

Data sharing is not applicable to this article.
